# “I see myself as part of the team” – family caregivers’ contribution to safety in advanced home care

**DOI:** 10.1186/s12912-018-0308-9

**Published:** 2018-09-14

**Authors:** Christiane Schaepe, Michael Ewers

**Affiliations:** Charité – Universitätsmedizin Berlin, corporate member of Freie Universität Berlin, Humboldt-Universität zu Berlin, and Berlin Institute of Health, Institute of Health and Nursing Science, Berlin, Germany

**Keywords:** Qualitative research, Family caregivers, Advanced home care, Home mechanical ventilation, Patient safety

## Abstract

**Background:**

The use of medical technology and the various contributing and interdepending human factors in home care have implications for patient safety. Although family caregivers are often involved in the provision of advanced home care, there is little research on their contribution to safety. The study aims to explore family caregivers in Home Mechanical Ventilation (HMV) safety experiences and how safety is perceived by them in this context. Furthermore, it seeks to understand how family caregivers contribute to the patients’ and their own safety in HMV and what kind of support they expect from their health care team.

**Methods:**

An explorative, qualitative study was applied using elements from grounded theory methodology. Data were collected through individual interviews with 15 family caregivers to patients receiving HMV in two regions in Germany. The audiotaped interviews were then subject to thematic analysis.

**Results:**

The findings shows that family caregivers contribute to safety in HMV by trying to foster mutual information sharing about the patient and his/her situation, coordinating informally health care services and undertaking compensation of shortcomings in HMV.

**Conclusion:**

Consequently, family caregivers take on considerable responsibility for patient safety in advanced home care by being actively and constantly committed to safety work*.*

Nurses working in this setting should be clinically and technically skilled and focus on building partnership relations with family caregivers. This especially encompasses negotiation about their role in care and patient safety. Support and education should be offered if needed. Only skilled nurses, who can provide safe care and who can handle critical situations should be appointed to HMV. They should also serve as professional care coordinators and provide educational interventions to strengthen family caregivers’ competence.

## Background

Advanced technology for the provision of enteral tube feeding, home-based dialysis, intravenous therapy and home mechanical ventilation (HMV) is widely used in the community in many western countries. Multiple factors have contributed to this converging trend, such as advances in technology, increased availability of ‘hospital at home’ services, demographic changes paired with an increasing number of people living with chronic conditions or surviving congenital conditions, reduced institutional care and cost savings [1, 2]. These developments enable technologically dependent patients with complex needs to remain at home while receiving intensive nursing care on a comparable level to that provided in the hospital setting [3]. Advanced home care, thus, promises to be a cost-efficient and patient-centered alternative to institutionalized care [4, 5].

This paper will focus on HMV as an example of advanced home care. The latter is a therapeutic option for individuals with various underlying diseases ranging from conditions leading to progressive respiratory failure to unsuccessful weaning after an acute respiratory failure. The HMV can be delivered noninvasively (via mask) or invasively (via tracheostomy) on a continuous or intermittent basis [6]. Users of HMV represent a vulnerable, heterogeneous, small, but increasing group of technology-dependent individuals in many western countries [7, 8]. Although the number of patients on HMV in Germany is unknown due to a lack of prevalence data, it is estimated that 20,000 individuals are living with HMV in Germany [9]. Caring for an individual receiving HMV is very complex, because it entails the care of a person who is receiving life support due to their critical illness and has substantial care needs. Their condition makes patients dependent on technological assistance and skilled nursing services providing personal care, several daily medical and therapeutic procedures, and educational and psychosocial support for the patients and their family.

The fact that ventilator-dependent patients receive up to 24-h professional nursing services and medical treatment in their private homes funded by the Statutory Health and Nursing Care Insurance in Germany is of particular relevance. The main goal of this form of advanced home care is to guarantee a hospital-like immediate and qualified intervention in life-threatening situations [10]. Thus, providing intensive care in a private home brings challenges for all actors involved, including health professionals, patients and family members [3]. For families, the intrusiveness of medical technology in the private home care setting and the constant presence of nurses, flanked by occasional visits of other health care providers, results in a lack of privacy, which often proves to be a great challenge [11–13].

Furthermore, advanced home care has implications for patient safety. Various contributing and interdependent human factors have an impact on patient safety in home care. The individual characteristics of the patients and their caregivers, the nature of health care tasks, the home and social environment, medical devices and new technology are major components [14, 15]. The home care setting, for example, has distinctive characteristics that are very different from institutional environments and that have an impact on patient safety. Home care nurses work in isolation and their role is rather that of a guest in the family’s home [15, 16]. The unique nature of each individual home contributes to home care being viewed as unregulated and uncontrolled [17]. Despite this background, corresponding research is mainly conducted in institutional settings and little attention has been paid to safety in home care [17, 18]. Recent research from Canada has gleaned information that adverse events in home care are not rare [19, 20]. The Pan-Canadian Home Care Safety Study reports that 10.1% of the clients experience adverse events annually and that 56% of these were predictable [21]. Existing studies on safety in home care focus on safety risks and specific adverse events, such as falls, pressure ulcers, unplanned hospital admissions and medication errors, which are reported from the perspective of the health care provider [18, 20, 22, 23]. Very few studies have, however, focused on patient safety in home care from the perspective of the patient and family caregiver [24, 25]. Among these studies, the Pan-Canadian Home Care Safety Study has found that patient safety is strongly influenced by the understanding of family members, caregivers and providers regarding safety [26]. Consistent with these findings, another Canadian study found that safety concerns from the perspective of patients and family caregivers are multidimensional and intersectional, and are influenced by physical, spatial and interpersonal factors [25]. That is also a reason why the general definition of *patient safety* needs to be broadened by incorporating the perspective of all actors involved, including the family caregivers [17, 27].

A recent scoping review found that the compulsory enrollment to take on the caregiver role, the lack of preparedness and support, and loss of control have an impact on family caregiver safety [28]. In addition, psychological and physical health impairments and financial problems create a safety concern for caregivers [28]. Whether this applies to advanced home care is not known. To date, most qualitative research on family caregiving in advanced home care focuses on the perspective of parent caregivers to children. These studies have shown that family caregivers play a pivotal role in advanced home care, providing complex caregiving tasks, including technical procedures in daily care. They advocate for their family members within the health care system, take care of the equipment and coordinate health care services [29–31]. The responsibility for care has been shifted from the personnel to the parents [12, 13, 32]. Consequently, physical and emotional burdens and social isolation among caregivers of technology-dependent children are widely reported throughout the literature [13, 31, 33]. The main concern of family caregivers regarding adult HMV users is the constant struggle with health care services, including the lack of involvement in decision-making processes, the lack of continuity of care and the inadequate professional support [34]. Accordingly, access to psychosocial support was reported as being important to family caregivers [35, 36].

Family caregivers might be the first to witness any safety-related issue in the home setting due to their daily interaction with the care recipient and the formers’ often extensive shared life experience, and can, consequently, provide a unique perspective of home health care delivery. Given that the number of patients who require advanced home care in general and HMV specifically will probably increase, there is a need to better understand the role of the various actors involved in patient safety in this context. Nevertheless, the literature on family caregiving and safety remains focused on two aspects. Family caregivers are either referred to as “secondary patients” who need to be protected from physical and emotional harm, or as easily available providers of care with the potential of harming their family members [37]. Family caregivers’ own perspective of their role in providing safety in home care has not yet gained attention from the research community. However, a better understanding of family caregivers’ perspective of their contribution to safety their perspective could provide health professionals with additional strategies for providing safe, effective and patient-centered care in the home setting. To date, little empirical work has been undertaken to examine family caregivers’ perspective of safety [28]. The present study, therefore, aims to fill this research gap by exploring family caregivers in HMV safety experiences and how safety is perceived by them in such a special care arrangement. Furthermore, it seeks to understand how family caregivers contribute to the patients’ and their own safety in HMV and what kind of support they expect from their health care team.

## Methods

An explorative, qualitative research design using elements of grounded theory methodology [38] has been chosen for this study.

The study was part of a larger, multistage qualitative health services research project called SHAPE (“Safety in Home Care for Ventilated Patients”) which aimed at providing impulses for the conceptualization of safety work in advanced home care based on empirical data from the perspective of both users and providers. Partial results of this study, which has been funded by the German Federal Ministry of Education and Research and was performed from 2013 to 2017, have been published elsewhere [39].

### Recruitment

Recruitment was facilitated by the staff of nursing care providers (gatekeepers) who are in daily contact with the families. They provided some basic verbal information and distributed an introductory letter about the study to eligible participants. Other ways of approaching participants were through a hospital-based specialized respiratory care center, a health care insurance company, personal contacts and organizations, such as the German Association for Muscular Dystrophy and patient advocacy groups. Those who were interested in participating contacted the research team themselves and or via the nursing care providers and a mutually convenient appointment for the interview was scheduled. Participants were recruited in rural and urban areas in Northeast and South Germany to identify regional differences.

Family caregivers had to be at least 18 years, speak and understand German, and be involved in the care of an adult HMV user in some way to be included in the study. Maximum variation in participant characteristics, for example age, relationship to the care recipient, years of experience of HMV in the home, was used as a sampling strategy. It aims to include a wide spectrum of participants to gain a broad insight into their diverse perspectives and experiences [40].

### Data collection

Data was collected on two visits, as part of an iterative process over a period of 12 months (from June 2014 to June 2015). Potential participants were given additional information prior to the onset of the study.

Written and oral informed consent to participation was obtained on the first visit. Participants were asked to provide sociodemographic information (e.g. age, hours of caregiving, income and educational level) and to fill in the Burden Scale for Family Caregivers [41]. In addition, sociodemographic-, disease- and treatment-related information of the patients was collected. It was made clear that participation was voluntary and that they could withdraw from the study at any point in the data collection or analysis. Participants’ confidentiality was guaranteed. On the second visit, a pilot tested, semi-structured interview guide with open-ended questions was used to elicit information on the everyday life of caregivers (“I would like to get an idea of how your everyday life looks like and therefore ask you to tell me how your day yesterday looked like”) and the role of HMV and their caregiving. They were further asked to give examples of situations where they felt particularly unsafe (“Can you describe a situation where you felt particularly unsafe?”), what they did in this situation, how the professionals reacted and what could have been done better or differently. At the end of the interview, they summed up their meaning of safety in home care. New questions evolved during data analysis and topics became more focused in later interviews.

Apart from a few exceptions, most of the interviews were conducted in the HMV recipient’s home. The interviews lasted between 32 and 250 min and were audiotaped (with one exception; permission was refused by one informant and detailed notes were recorded). Two researchers were present during the interviews in most cases. Nonverbal expressions and gestures, potential disruptions, and the topics addressed before and after the interview were recorded in an interview protocol. An additional, detailed observational protocol was written on the home environment.

### Data analysis

Although data collection and data analysis were intended to occur concurrently in an iterative process, this could not always be realized due to initial recruitment difficulties. The interviews were transcribed verbatim and identifying information were pseudonymized in this process. The analysis was performed in German and the software MAXQDA 11 (verbi GmbH, Berlin Germany) was used to organize and manage the data. The thematic analysis began after the first interview with repeated reading of the first transcripts in order to become immersed in the data [42–44]. In the next step, the data were coded. Three forms of coding were employed*: Open coding* with in vivo coding was performed. The constant comparison technique was used with codes and concepts and clustered to create preliminary categories. Connections between categories were built in the *axial coding*. In *selective coding*, categories were saturated with data from new interviews. Memos were written throughout the whole analysis process to document ideas and reflections about the emerging codes and categories. After all the data were coded, the categories were sorted and combined into themes. Finally, several relevant themes were defined and named and condensed for reporting.

### Trustworthiness

Strategies that were used to evaluate the rigor of the study were based on the concept of trustworthiness by Lincoln and Guba [45]. Credibility was strengthened by the prolonged engagement in the field and by maximum variation sampling [46]. Prolonged engagement means that the researcher spent extended time in the field in order to gain a deeper understanding of the social context of the interviewees’ narratives, which helped to gain their trust and thereby facilitated authentic data collection. Dependability was enhanced by performing the analysis as part of a research team. To this end, several discussions and reflections were done throughout the analysis process. The team discussed and reflected for example on alternative ways of approaching participants in order to avoid selection bias, if more variation was needed in the sampling, the next analytical steps that had to be taken, the themes that emerged from the data. A thick description of the sample, setting and data collection, and analysis are presented for the reader’s judgment of transferability.

## Results

### Sample description

A total of 15 relatives of HMV patients gave consent to participate in the study (see Table 1 for participants key characteristics). Nine of them are spouses or partners, three mothers, two children and one sister. The participants’ age ranged from 31 to 83 years, with three males and 12 females. Four caregivers were employed, eight retired and three partially retired or unemployed. Eight of the 15 participants were living in a common household with the HMV users and seven were living separately.

**Table 1:** Characteristics of family caregivers and care recipients

Caregivers Pseudonym	Age of caregiver	Gender of caregiver	Level of Education	Employment status	Relationship to the care recipient	Living arrangement	Years of experience of HMV in the home	BSFC Results*	Care recipients Disease group	Hours of HMV	Hours of nursing service	IV or NIV
Mrs Becker, Katrin	31	Female	High School	half-time employment	daughter	separated from the patient	1	40	neuromuscular disorder	continuous	24hs	IV
Mrs Wagner, Monika	63	Female	College or University	part-time retirement	wife	together with the patient	2	13	neuromuscular disorder	continuous	24hs	IV
Mrs Yilmaz, Fatma	60	Female	High School	full-time employment	wife	together with the patient	7	23	neuromuscular disorder	continuous	24hs	IV
Mr Meyer, Peter	56	Male	College or University	early retirement	son	separated from the patient	10	27	pulmonary disease	continuous	24hs	IV
Mrs Wolf, Christa	71	Female	Basic	retirement pension	wife	together with the patient	1	16	neuromuscular disorder	> 16hs	24hs	IV
Mr Richter, Karl	79	Male	College or University	retirement pension	husband	separated from the patient	missing	missing	vegetative state	> 16 hs	24hs	IV
Mrs Bauer, Ursula	70	Female	High School	retirement pension	mother	together with the patient	41	19	tetra paresis	< 16 hs	9hs	IV
Mrs Braun, Sabine	56	Female	Basic	early retirement pension	wife	together with the patient	6	21	Tetra paresis	< 16 hs	without nursing service	IV
Mrs Schulz, Angelika	62	Female	Basic	half-time employment	sister	separated from the patient	4	28	metabolic disease	continuous	24hs	IV
Mrs Werner, Gabrielle	54	Female	High School	full-time employment	wife	separated from the patient	3	23	neuromuscular disorder	continuous	24hs	IV
Mrs König, Renate	61	Female	High School	early retirement pension	spouse	separated from the patient	1	37	neuromuscular disorder	continuous	24hs	IV
Mrs Peters, Birgit	59	Female	High School	no gainful employment	mother	together with the patient	41	7	neuromuscular disorder	continuous	8hs	NIV
Mrs Koch, Ingrid	69	Female	Missing	retirement pension	wife	together with the patient	missing	missing	infectious disease	continuous	8hs	IV
Mrs Zimmermann, Andrea	56	Female	Basic	unemployment	mother	together with the patient	15	45	tetra paresis	10hs	11hs	NIV
Mr Hoffmann, Günther	83	Male	High School	retirement pension	husband	together with the patient	14	31	neuromuscular disorder	continuous	24hs	IV

*BSFC Results: no/little burden: 0–24 points, moderate burden: 25–55 points, severe burden: 56–84 points

The nature of family caregivers’ involvement in everyday care varies. While some of them provide 24-h care (including endotracheal suctioning, supervision of the functioning of the technical devices and constant vigilance over the care recipient), others merely visit the patients in their homes on a regular basis. The degree of involvement ranged from 1, 5 to 24 h per day. All but one family were receiving (professional) nursing services. The extent of skilled nursing care offered ranged from 8 to 24 h per day. Despite this variation, the results of the Burden Scale for Family Caregivers in our sample show that most of the participants experience little and moderate burden (see Table 1).

Apart from using HMV, the care recipients are similarly a heterogeneous group. The reasons for HMV dependency varied from neuromuscular diseases, restrictive, thoracic disorders to chronic obstructive pulmonary disease. Average daily ventilation use ranged from 10 to 24 h. A more detailed description of the care recipient’s characteristics can be found elsewhere [39].

### Contribution of family caregivers to safety in HMV

Several themes emerged from the interview data during analysis exploring the broad spectrum of safety experiences and perspectives of relatives of HMV users. It also became apparent that family caregivers of ventilated patients use several strategies to cope with their specific situation and to guarantee the care recipients’ and their own safety. “Fostering mutual information sharing about the patient and his/her situation”, “coordinating health care services” and “compensating for shortcomings in HMV” are the most evident contributions family caregivers make to guarantee safety in advanced home care for technologically dependent patients based on our empirical data.

### Mutual information sharing

Family caregivers in this study often try to foster mutual information sharing about the patient and his/her situation based on their familiarity and their intimate knowledge about their relatives’ needs, wishes and personal preferences. That is particularly the case when the patients themselves have limited communication possibilities due to the ventilation or when they cannot express themselves due to their vulnerable physical or mental status. They not only intend a more personalized care by sharing their information with members of the health care team, but rather to prevent adverse events and promote patient safety. Exemplarily, this strategy is being applied by Ms. Yilmaz, who has been caring for her ventilated and bedbound husband 24-h a day for many years. Due to her long-standing marriage and her extensive experience of caring for her technologically dependent husband, she is convinced that she knows his needs, wishes and preferences very well. She wants to share this unique information with the nurses so that they can act accordingly. In exchange, she herself wants to be informed regularly about what happens in everyday care and how her husband reacts to the care services offered. The following quotation illustrations her motivation:

*“Well, I see myself as part of the team, I would say, I do other things, but anyway. However, if this exchange happened more often, my husband would be or feel better. If he was better, then that would mean safety for me.*” (Ms. Yilmaz).

Although Ms. Yilmaz is aware that she is not performing the same duties as the nurses, she perceives herself as a constitutive member of the care team. Regular information exchange between family caregivers and the health care team about the patients’ needs, wishes and preferences would, according to her assumption, benefit the patient’s health and, thus, promote safety for all parties involved.

Many other family caregivers from our sample wish to be seen as a relevant source of information about the patients and, therefore, get more involved in caring for their loved ones, albeit to a varying extent. Family caregivers wish to be taken seriously so that they can speak for the care recipient and offer insights into their individuality. However, this mutual information sharing is not always valued, and some health professionals make the family caregivers feel like they are an unwanted factor in HMV. In such cases, decisions regarding the patient are made without them, their opinion and experience is deemed insignificant, their perspective is not heard, and information is withheld. Feelings of insecurity on the side of the users, or even worse, near misses and adverse events are consequences that might arise from this disregard of the family caregivers and the information they have to share in advanced home care.

### Informal coordination

Family caregivers contribute to safety in HMV by coordinating care. This is not a formal function assigned to them, it is rather imposed on them accidentally. However, this implies a substantial organizational effort and is sometimes a burden for them. They identify what equipment is needed for the provision of care (e.g. wheelchair, second back-up ventilator, consumable materials, care aides) and make sure it is available in time. Occasionally, they have to negotiate with the health insurance company in an attempt to gain access to fully functional replacement devices or other equipment on site. Moreover, family caregivers sometimes perceive the need to link and coordinate the activities of the several isolated working health professionals involved in HMV. This is demonstrated by the experiences of Ms. Becker. Although she is not living together with her 24-h a day ventilated father, she is still actively involved in his everyday care:

*“Well, I am also the link between the therapists, physicians, nurses and suppliers of care equipment. I am often present, so that I know what is being said, so that I can transfer this to everybody. I am part of this.” (Ms. Becker).*

This citation shows that Ms. Becker takes on the responsibility of bringing together the different health care providers involved in the home care of her father. She is the one who transmits information among them, which otherwise would not have been transmitted, which might cause severe safety problems. This requires her presence when the health professionals are doing home visits and to remember all the appointments of the different parties involved. She also keeps an information diary, where she expects the health professionals to write to her when something unexpected occurred.

This role of an informal care coordinator is not only very responsible, but also an exhausting one for the family caregivers. Sometimes they find themselves between the different sides, especially when some parties are withholding information from them or each other. If the family caregivers are actively excluded from the team, feelings of uncertainty, worry and anger are triggered.

### Compensating for shortcomings

Experiences with professional home nursing services differ widely. Those who have positive experiences can rely completely on nurses in terms of safety. Others who have had negative experiences (e.g. when nurses fall asleep during the night shift) put little trust in them and want to be prepared for compensating of shortcomings in HMV.

Some family caregivers seek to expand their knowledge and skills in order to ensure a high degree of safety for the patients using various strategies. Some report having been instructed by nurses, while others have learned by observing nurses performing the tasks. When they are not instructed regarding care and emergency situations, it is not uncommon that they try to acquire skills behind the nurse’s back in order to be prepared. The elderly married couple Mr. and Ms. Bauer who are taking care of their ventilated and multi-morbid adult son can be seen as an example of that strategy:

*“What I have also done, yes, is that I have changed the cannula myself together with my husband. I said I would simply like to do it, because I have to be able to do it in an emergency.” (Ms Bauer).*

The context of this citation suggests that neither Ms. nor Mr. Bauer have been taught how to change a tracheostomy tube, although they would like to know how to do it so that they can handle critical situations themselves when the professionals are not observing or available. The Bauers – like other family caregivers in HMV – want to be prepared for handling emergency situations, but are prevented from doing so.

Most of the family caregivers in our study tried to keep control over the home care situation, making sure that the care recipient is well cared for and nothing is overlooked. Some of them reported that they had to remind the nurses of different nursing measures, such as changing the tube or administrating medication. Some family caregivers, such as Ms. Zimmermann, even try to instruct the nurses to ensure the HMV recipient’s safety in the absence of a proper initial on-the-job training for new and inexperienced nurses. She cares for her adult son during the daytime, whereas a nurse is on duty and responsible for his care and safety at night when he is mechanically ventilated. However, Ms. Zimmermann is constantly alert.

*“I instruct them always. I, I as mother, have to instruct qualified personnel, show them how to catheterize, I have to do it, that isn’t my job.” (Ms. Zimmermann).*

Ms. Zimmermann is well aware that instructing or supervising professional caregivers is not her task as a mother. She must do it anyway and compensate for qualification deficits as well as organizational shortcomings so that her son gets proper help when necessary during the night.

Some family caregivers even feel the need to prepare themselves and the health care team for emergency situations. Exemplarily, Mr. Hoffmann’s wife cannot move or breathe by herself because of her advanced neuromuscular disease and so she is completely dependent on the medical devices and human assistance. Mr. Hoffmann simulates critical situations like a power failure and observes the nurses’ reactions:

*“And you know, my presence is necessary. The women are not able to do it alone. I understand that, nervous, making mistakes and then this and that happens. And you have to have that under control. The more you train, the better it is.” (Mr. Hoffmann).*

Mr. Hoffman guides the “training” to make sure that everybody on the care team is prepared for a potentially hazardous situation. Thereby, he is the one who tries to gain control in order to prevent potential risks for adverse events. The citation further illustrates the shift of roles: He is in charge, guiding the training and not the nurses, as it should be from a professional point of view.

Not all family caregivers in our study might go as far as Mr. Hoffmann. However, most of them are on alert and constantly on call for supervision. They need to be sure that the ventilated care recipient is monitored closely and that someone can intervene quickly at any time. When nurses perceived as inexperienced or insecure are in charge, relatives feel indispensable and make arrangements to be at home to supervise the care recipient and the functioning of the technical devices themselves. Being present enables family caregivers to intervene if necessary. As a result of their feeling indispensable for the patient’s safety, some family caregivers mentioned not having taken time off for many years and being trapped in their own house. However, they are convinced that they make an important contribution to patient safety in advanced home care by undertaking this form of compensation.

## Discussion

Moving advanced medical technology from institutional settings to the community equates partially with a shift of responsibility for patient care from professionals to family caregivers [13]. Even if nurses are responsible for advanced home care up to 24 h day, such as in Germany, family members still have an active, complex and demanding part to play. The findings of this study extend previous research by showing that family caregivers take considerable responsibility for patient safety by being actively and constantly committed to safety work. This is in line with the findings of the Pan-Canadian Home Care Safety Study stating that all actors (clients, family members, caregivers and paid providers) in home care are creating and maintaining safety [26]. Moreover, the findings of the present study broaden the body of literature on family caregivers and safety by indicating that in many cases, family members are the ones to ensure patient safety in advanced home care by applying several strategies. Exemplarily, their intimate knowledge of the needs, wishes and preferences of the care recipient is a valuable resource for the health care team and family care givers are acting a guarantor of patient safety by sharing this information. Therefore, a change in focus from considering family caregivers as “secondary patients” or harmful to patients [37] to acknowledging their valuable contribution to patient safety in HMV is needed. Raising awareness about family caregivers valuable contribution to patient safety among nurses and other health professionals and conducting further research on family caregivers’ contribution to safety would be ways towards such a change.

However, the study findings illustrate that some of the family caregivers’ actions are putting the client at risk, for example, when relatives whose need for preparedness is not met through proper professional instructions execute advanced nursing tasks, such as changing the tracheostomy tube behind the nurse’s back or simulating emergency situations. Their intention is certainly not to put the care recipient in danger, but they need to be sure that they can offer immediate assistance in life-threatening situations when professionals cannot. Our findings show that even qualified nurses sometimes lack expertise regarding HMV care, which makes family members feel indispensable and responsible, as reported in previous studies [34, 47]. Therefore, they feel forced to gain knowledge and technical skills regarding HMV therapy to compensate for this lack of professional expertise. It is problematic that family members must take the initiative to (re)gain control, instead of the nurses enabling them to handle critical situations and strengthening their self-management competence through educational interventions.

### The partnership approach

The need for family members to participate in the provision of home care has been highlighted before in research on technologically dependent children [12, 13], but has not been discussed previously either in relation to family caregivers of adult patients or to safety. The current findings indicate that family caregivers are involved in care, even if qualified nurses are in charge. Furthermore, they feel responsible and indispensable for the safety of their loved ones, as reported previously [34]. This perceived responsibility also entails supervising and educating nurses in the management of the devices, as seen in previous studies [32]. Family caregivers feel forced to take on these tasks because they do not feel that the nurses in charge are sufficiently prepared to care for the HMV recipient properly. Similar to previous findings [34], they compensate for their health care professionals’ perceived lack of competence by being present and constantly alert. However, participation in care should not equate with compensating for health care providers’ deficiencies or training professionals, but valued as an important resource. The rationale for their need to participate in the provision of home care is that they see themselves as patients’ advocates due to their closeness to their loved ones and due to their long caring experience, which agrees with other studies [31, 48]. This valuable perspective helps to identify issues that professionals may not recognize and, therefore, foster patient safety.

Our study further shows that being involved in HMW means knowing that the loved one is well cared for and makes the family caregivers themselves feel safer. That patient safety is inextricably linked with family caregivers’ safety has even been found in previous research [17]. Therefore, advanced home care draws attention to the partnership approach between health professionals and family caregivers. So far, caregiver roles and responsibilities have not been clarified and should, therefore, be openly negotiated between partners and not be imposed upon families against their will [49]. Due to the complex nature of caring for this high-risk population and the numerous professionals involved in advanced home care, negotiation of roles might be even more relevant in this setting than in usual home care. It also seems important that this role negotiation does not result in an over-reliance on family caregivers to keep the patient safe, this being the key role of contemporary nursing [50].

### Family caregivers should be offered support

The results of the Burden Scale for Family Caregivers show that most participants in our sample experience some burden due to their participation in home care provision, although in most cases qualified nurses are in charge up to 24 h a day, which could be expected to ease the burden. One explanation might be that they cannot rely completely on the health professionals providing safe and quality care. As a consequence of their lack of trust in professional care, and mutual information sharing as well as difficulties with health care team coordination, they are concerned about the patients’ safety and feel forced to partially take on professional roles and responsibilities which can be burdensome. Thus, it is of utmost importance that they have permanent access to professional problem-solving support along with psychosocial and emotional support, which echoes previous studies about technology-dependent patients’ close relatives, emphasizing the importance of the availability of professional support either in-person or by phone [31, 36, 51, 52]. It is imperative that nurses and other health professionals acknowledge the relatives’ perception of their own support needs, offer targeted support themselves or refer them to relevant services. The support should be easily available to promote a sense of safety. This support is particularly important at the beginning of HMV [36]. Furthermore, it is equally important that family caregivers are educated on how to handle unexpected situations and properly supervised to protect them from becoming a risk factor for the care recipient. This is a key nursing task, which is apparently not fulfilled sufficiently in HMV in Germany.

#### Limitations

The strength of this study lies in integrating the family caregivers’ voice into home care safety research. At the same time, it is its major limitation that these findings only reflect a single perspective. Future studies triangulating our findings with the perspectives of patients and nurses or other professionals need to be undertaken to provide a holistic understanding of patient safety in home care.

As in all qualitative studies, findings are context-bound. The care provision of HMV in Germany is different to that in other countries and it remains unclear whether family caregivers’ contribution to safety is dependent on the qualification of formal caregivers. It is, however, likely that the findings will have some relevance for other family caregivers in advanced home care, which could be investigated in further research.

Furthermore, recruiting participants through nursing service providers can be a disadvantage, because these might have selected relatives who are satisfied with their services. However, further recruitment strategies were used to address this risk of selection bias (see above).

## Conclusions

As advanced home care is gaining momentum, there is an increasing need to focus on patient safety in this setting. The perspective of family caregivers presented yields interesting insights into the multiple tasks family caregivers take on to guarantee safety for their loved ones. In conclusion, nurses and other health professionals should meet family caregivers with respect and value their considerable role and the responsibility that they take for patient safety. Given the essential role they play in advanced home care, family caregivers should be seen as important, valuable and trustworthy partners.

However, the fact that family caregivers are performing nursing and medical tasks and even train professionals raises serious concerns. Instead, only competent personnel should be in charge of helping family caregivers feel less indispensable for patient safety. Nurses and other health professionals should act in partnership with family caregivers and allow them to deliberately choose their role in patient care and safety.

### Implications for nursing practice

Given family caregivers’ enormous commitment to ensure patient safety, nurses need to regain their professional responsibilities and duties from families. Our findings suggest that only skilled nurses who can provide safe care and handle critical situations should be appointed for HMV. Nurses should also serve as professional care coordinators and provide educational interventions to strengthen families’ competence. An adequate training should encompass providing concrete instructions on areas in which family caregivers would like to be involved, such as the proper use of the medical equipment or preparedness for emergency situations. Another important family nursing intervention should be the negotiation of roles in advanced home care.

## Abbreviation

HMV: Home mechanical ventilation
